# An Anti-VEGF-B Antibody Reduces Abnormal Tumor Vasculature and Enhances the Effects of Chemotherapy

**DOI:** 10.3390/cancers16101902

**Published:** 2024-05-16

**Authors:** Peter W. Janes, Adam C. Parslow, Diana Cao, Angela Rigopoulos, Fook-Thean Lee, Sylvia J. Gong, Glenn A. Cartwright, Ingrid J. G. Burvenich, Ulf Eriksson, Terrance G. Johns, Fiona E. Scott, Andrew M. Scott

**Affiliations:** 1 Tumour Targeting Program, Olivia Newton-John Cancer Research Institute, Heidelberg, VIC 3084, Australia; 2 School of Cancer Medicine, La Trobe University, Melbourne, VIC 3083, Australia; 3 School of Computing, Engineering and Mathematical Sciences, La Trobe University, Melbourne, VIC 3083, Australia; 4 Department of Molecular Imaging and Therapy, Austin Health, Melbourne, VIC 3084, Australia; 5 Division of Vascular Biology, Department of Medical Biochemistry and Biophysics, Karolinska Institute, 171 77 Solna, Sweden; 6 Oncogenic Signalling Laboratory, Telethon Kids Cancer Centre, Telethon Kids Institute, Nedlands, WA 6009, Australia; 7 Medical School, University of Western Australia, Crawley, WA 6009, Australia; 8 Department of Medicine, University of Melbourne, Parkville, VIC 3052, Australia

**Keywords:** tumor angiogenesis, antibody, therapeutic, vessel normalization

## Abstract

**Simple Summary:**

In order to grow, tumors need nutrients and oxygen, which are supplied by new blood vessels that are often abnormal and leaky. The development of these blood vessels is driven by proteins called growth factors, which bind to receptor proteins on other cells. We show for the first time that an antibody against a vascular growth factor called VEGF-B, when used to treat mice with tumors, reduces the amount of abnormal tumor blood vessels, and inhibits tumor growth, as well as improving the response to chemotherapy. These results suggest that targeting VEGF-B could be a potential approach for anti-cancer therapy, particularly in combination with chemotherapy.

**Abstract:**

The vascular endothelial growth factors (VEGFs) and their receptors (VEGFRs) are key regulators of blood vessel formation, including in tumors, where their deregulated function can promote the production of aberrant, leaky blood vessels, supporting tumor development. Here we investigated the VEGFR1 ligand VEGF-B, which we demonstrate to be expressed in tumor cells and in tumor stroma and vasculature across a range of tumor types. We examined the anti-VEGF-B-specific monoclonal antibody 2H10 in preclinical xenograft models of breast and colorectal cancer, in comparison with the anti-VEGF-A antibody bevacizumab. Similar to bevacizumab, 2H10 therapy was associated with changes in tumor blood vessels and intra-tumoral diffusion consistent with normalization of the tumor vasculature. Accordingly, treatment resulted in partial inhibition of tumor growth, and significantly improved the response to chemotherapy. Our studies indicate the importance of VEGF-B in tumor growth, and the potential of specific anti-VEGF-B treatment to inhibit tumor development, alone or in combination with established chemotherapies.

## 1. Introduction

Angiogenesis, the organized growth and remodeling of the vascular system, involves a complex collection of growth factors and receptors. The best characterized of these is the vascular endothelial growth factor (VEGF) family. The mammalian VEGF family consists of five proteins: VEGF-A, VEGF-B, VEGF-C, VEGF-D (also known as FIGF) and placenta growth factor (PlGF). These members differ in expression pattern, specificity and biological function [1]. The VEGF family members are homodimeric polypeptides, although naturally occurring heterodimers of VEGF-A and PlGF have been described [2]. Their function is mediated by their specificity for three endothelial receptor tyrosine kinase receptors (VEGFR-1, VEGFR-2 and VEGFR-3) and two co-receptors (Neuropilin-1 and -2). VEGF-A binds to both VEGFR-1 and VEGFR-2, whereas PIGF and VEGF-B bind to only VEGFR-1. VEGF-C and VEGF-D bind to VEGFR-2 and VEGFR-3, and also the Neuropilin co-receptors [3].

Angiogenesis is critical not only in normal development but also in disease, including eye diseases and prominently in cancer, where hypoxia-driven angiogenesis supports tumor growth and spread. The importance of VEGF signaling in tumor angiogenesis is well established, promoting survival, proliferation, sprouting, migration and tube formation of endothelial cells [3]. Pathological angiogenesis involves persistent and unresolved signaling, resulting in increased vascular permeability, where leaky, abnormally dilated tumor vessels form, increasing intra-tumoral pressure and metastasis, and decreasing accessibility to chemotherapy and immune-mediated anti-tumor responses [3,4]. The first approved VEGF-targeted agent was the anti-VEGF-A monoclonal antibody bevacizumab (Avastin), for metastatic colorectal cancer (mCRC), for which it showed clinical benefit in patients when combined with chemotherapy [5,6]. Bevacizumab is now approved for multiple cancer types, including lung cancer (NSCLC), renal cell carcinoma, liver, ovarian and cervical cancer, and glioblastoma (GBM) [6]. Many patients treated with bevacizumab, however, re-establish angiogenesis despite VEGF-A inhibition [7]. Additionally, Aflibercept targets VEGF-A, VEGF-B and PlGF, and has been approved in metastatic colon cancer. Various agents targeting VEGF receptors have also been approved, including tyrosine kinase inhibitors and the VEGFR2-specific antibody Ramucirumab [8]. Although much focus has been given to VEGF-promoted angiogenesis through VEGFR-2, studies have also suggested the importance of VEGFR-1 in pathological angiogenesis [3]. To date, no antibodies specifically targeting VEGFR1 or its selective ligands VEGF-B and PlGF have been approved, despite the initial promise of PlGF antibodies [3].

VEGF-B, discovered after VEGF-A and PlGF, remains the least well characterized of the ligand family. Gene knockout studies have found that VEGF-B is not essential for the growth and development of the peripheral vascular system but has a role in the normal development of the coronary vasculature and in normal physiologic responses to ischemia and vascular occlusion [9,10,11]. Interestingly, VEGF-B is co-expressed with nuclear genes encoding mitochondrial proteins, suggesting a role in metabolism, and it stimulates the endothelial cell expression of fatty acid transporter proteins (FATPs), facilitating the trans-endothelial transport of fatty acids [12]. VEGF-B promotes the uptake of fatty acids into podocytes in the kidney leading to lipotoxicity, podocyte loss, proteinuria and diabetic nephropathy [13]. Accordingly, targeting VEGF-B in models of type 2 diabetes has demonstrated improved insulin sensitivity, glucose tolerance and disease stabilization [14].

VEGF-B is increased in many malignant tissues including colorectal, ovarian, renal cell and prostate cancer [15,16]. In HER2-expressing epithelial breast cancers, VEGF-B expression has been associated with reduced disease-free survival and overall survival rates in patients who are lymph-node-positive [17]. Stimulation of the VEGFR-1 receptor with VEGF-A or VEGF-B was shown to promote tumor cell migration and increase invasive potential in colorectal cancer [18], and VEGF-B expression is associated with metastasis and poor prognosis in lung squamous cell carcinoma and melanoma [19]. VEGF-B dysregulation has also been correlated with microvascular density in oral squamous cell carcinoma [20] and with advanced stage, tumor multiplicity and vascular invasion in hepatocellular carcinoma [21].

A monoclonal antibody specific for mouse and human VEGF-B (2H10) was previously generated, displaying inhibitory activity in vitro [22]. We now show biological effects of 2H10 in mice, indicating changes in tumor vascularization, inhibition of tumor growth, and enhanced response to chemotherapy, suggesting potential as a VEGF-B targeted therapy.

## 2. Results

### 2.1. VEGF-B Is Expressed in Tumor Stroma, Vasculature and in Tumor Cells in a Range of Tumor Types

**Figure 1:**
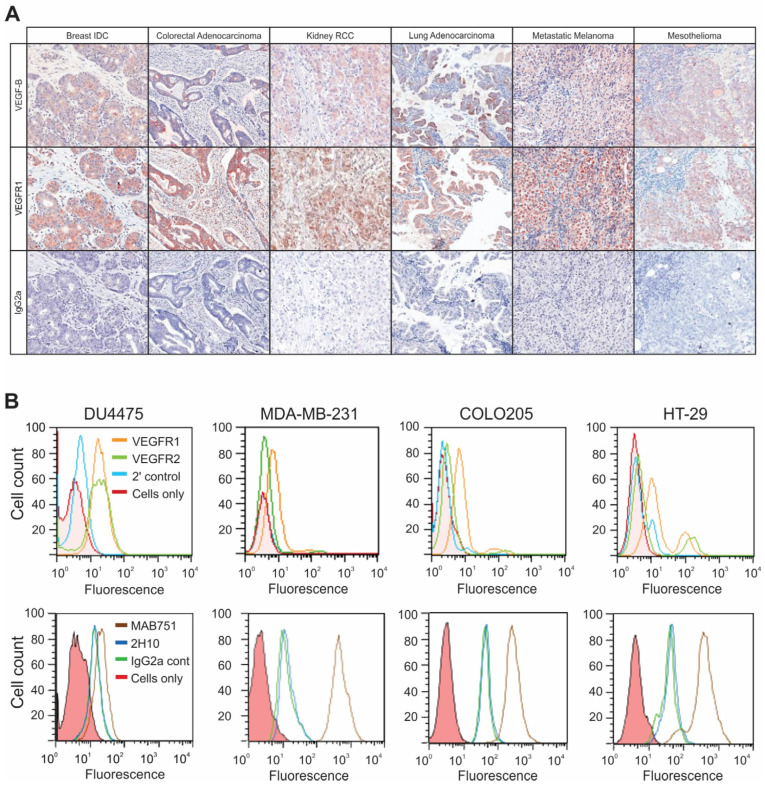
VEGF-B and VEGFR1 are widely distributed across human tumor types. (**A**) Immunohistochemical staining of VEGF-B, by 2H10, and VEGFR1 (Thermo Scientific) was assessed in formalin-fixed paraffin-embedded sections of eleven human tumors and ten normal human tissues. Representative images of samples from breast intraductal carcinoma, colorectal adenocarcinoma, renal cell carcinoma, lung adenocarcinoma, metastatic melanoma and mesothelioma are provided. Staining was observed within tumor cells, vessels and stroma. Images are shown at 20×. Non-specific staining was not observed with the IgG2a isotype control murine antibody. (**B**) Top row: cell surface expression of VEGFR-1 (orange) and VEGFR-2 (green), was explored in four cancer cell lines (DU4475 MDA-MB-231, COLO205, and HT29) by antibody staining and flow cytometry analysis. Staining was compared with secondary antibody alone (blue) or unstained cells (red). Bottom row: VEGF-B expression was explored in the same cancer cell lines. Cells were permeabilized before staining with MAB751 (brown) and 2H10 (blue). Controls included murine IgG2a antibody alone or unstained cells, as indicated.

The expression of VEGF-B, and its receptor VEGFR-1, was explored across a panel of eighteen normal human tissues and fifteen common tumor types (breast intraductal carcinoma, breast intralobular carcinoma, mesothelioma, melanoma, colorectal adenocarcinoma, renal cell carcinoma, lung adenocarcinoma, lung squamous cell carcinoma, hepatocellular carcinoma, pancreatic adenocarcinoma, prostate, ovary and uterine adenocarcinoma, bladder transitional cell carcinoma, and brain glioblastoma multiforme) from 12 to 15 different human donors using tissue microarrays (TMAs). Anti-VEGF-B antibody 2H10 and anti-VEGFR-1 staining was evident in both tumor and stromal cells (Figure 1A). The percentage of positive 2H10 antibody staining was comparable to that with a commercial anti-VEGF-B antibody (MAB751, R&D Systems); both VEGF-B antibodies showed cytoplasmic staining of tumor and normal tissues, apart from in the brain where 2H10 only bound tumors (4/10) versus normal (0/12), while MAB751 bound tumors (10/10) and some normal samples (4/13) (Tables S1–S3).

The assessment of appropriate tumor cell lines amenable to anti-VEGF-B therapy was performed by flow cytometry following immunofluorescent staining of cell surface VEGFR-1 and VEGFR-2, or intracellular VEGF-B expression in breast carcinoma (MDA-MB-231 and DU4475, MCF-7) and colon carcinoma cell lines (COLO205, HT29, LIM1215, LIM1899, SW1222) (Figure 1B). All cell lines examined stained for intracellular VEGF-B with MAB751, to varying degrees. In contrast, staining with 2H10 was equivalent to the IgG2a isotype control. The lack of specific 2H10 binding to cells may reflect its known binding to a conformational epitope [22], which may be lost in ethanol-fixed, permeabilized cells in vitro. In contrast, MAB751 binds denatured VEGF-B (e.g., by Western blot), so it is likely less conformation-dependent. Cell surface VEGFR-1 and VEGFR-2 expression was detected on the majority of cell lines, but only weakly on MDA-MB-231 (Figure 1B) and MCF-7 cells. The widespread distribution of VEGF-B and its receptor VEGFR-1 across multiple tumor tissue types and cell lines suggests that many tumors could be amenable to anti-VEGF-B antibody therapy.

### 2.2. Anti-VEGF-B 2H10 Targets VEGF-B in Tumors In Vivo

**Figure 2:**
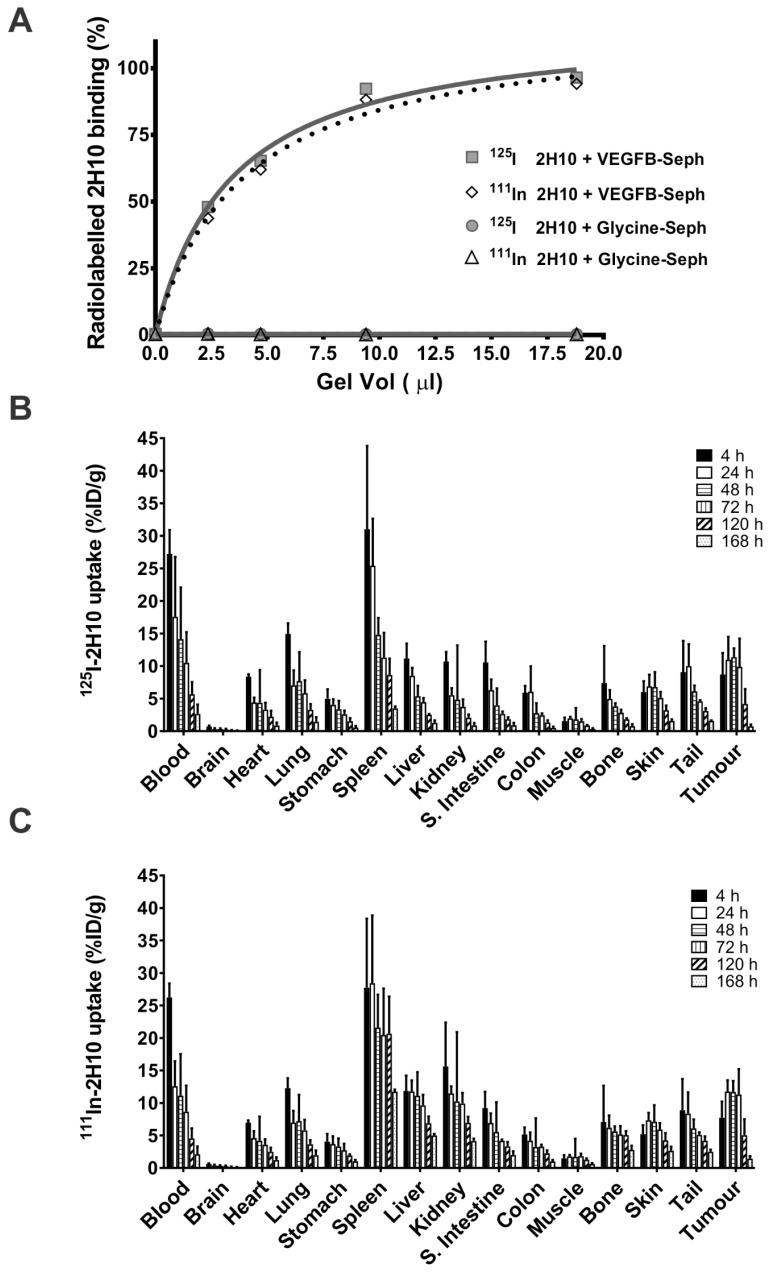
Immunoreactivity and tissue distribution of radiolabeled murine 2H10 antibody. (**A**) Binding to increasing quantities of VEGF-B-conjugated Sepharose or control glycine-conjugated Sepharose to measure specific and non-specific binding of ^125^I-murine 2H10 and ^111^In-murine 2H10. (**B**,**C**) Biodistribution of (**B**) ^125^I-2H10 and (**C**) ^111^I-2H10 in mice bearing DU4475 tumors. Six groups of 5 mice were injected with ^111^In-labeled murine 2H10 antibody (4.5 µg, 17.8 µCi) or ^125^I-labeled murine 2H10 antibody (4.8 µg, 7.3 µCi), and tumors and tissues were recovered for analysis from 4 to 164 h post injection.

To examine its tumor specificity in vivo, 2H10 was radiolabeled with either ^125^I or ^111^In at specific activities of 1.1 mCi/mg and 3.3 mCi/mg, respectively. The immunoreactivities of the two radioconjugates were determined using a binding assay in the presence of excess antigen in the form of VEGF-B-conjugated Sepharose beads, compared to control glycine-conjugated Sepharose, demonstrating the specificity of antibody binding (Figure 2A). The immunoreactivity of both ^125^I-2H10 and ^111^In-CHX-A”-DTPA 2H10 remained high, (94.2–96.5%), while binding to control glycine-conjugated Sepharose was negligible. The stability of the murine antibody radioconjugates in human serum (37 °C for 8 days) was also similar, decreasing to 70–72.9%. The radiochemical purity of the radioconstructs was retained at >96% over the 8 day incubation.

Biodistribution of ^111^In- and ^125^I-labeled murine 2H10 was analyzed in mice bearing DU4475 xenografts in a time course from 4 to 168 h following injection of a mixture of trace doses of ^125^I-2H10 (4.8 µg, 7.3 µCi) (Figure 2B) and ^111^In-2H10 (4.5 µg, 17.8 µCi) (Figure 2C). The uptake (% injected dose) of labeled 2H10 peaked in DU4475 tumors from 24 to 72 h, while levels in normal tissues were typically highest at 4 h before rapidly decreasing, indicating preferential retention in tumors and relatively low non-specific distribution in normal tissues. However, there was high uptake in the spleen, where ^111^In-2H10 in particular remained relatively high up to 120 h, possibly reflecting Fc-mediated binding of the murine IgG2a to splenocytes. To investigate this, we compared the biodistribution of a ^131^I-labeled murine IgG1 version of 2H10 with the ^125^I-labeled murine IgG2a 2H10. High immunoreactivities of both ^125^I-2H10 (IgG2a) and ^131^I-2H10 (IgG1) to antigens was conserved (96.5% and 91.6%, respectively). Antibodies were injected alone (Figure S1A,B) or with 1.1 mg unlabeled 2H10 (murine IgG2a) as a cold competitor (Figure S1C,D). A mixture of both radiolabeled 2H10 mAb isotypes was administered to non-tumor-bearing mice, and biodistribution was measured at 3 h (Figure S1A,C) and 24 h (Figure S1B,D). Only the radiolabeled IgG2a and not the IgG1 version of 2H10 bound strongly to the spleen at 3 h (Figure S1A, IgG2a = 38.50 ± 11.07; IgG1 = 7.86 ± 1.21) and 24 h (Figure S1B, IgG2a = 18.59 ± 7.63% ID/g; IgG1 = 5.90 ± 0.71%ID/g) following injection. The higher IgG2a splenic uptake was decreased to the IgG1% injected dose (ID)/g levels by the co-administration of unlabeled 2H10 at 3 (Figure S1C) and 24 (Figure S1D) hours. This indicates that binding of 2H10 to mouse spleen is due to known Fc interaction of IgG2a to splenocytes rather than Fv binding to antigens.

### 2.3. Anti-VEGF-B 2H10 Reduces Tumor Growth and Alters Tumor Vasculature

**Figure 3:**
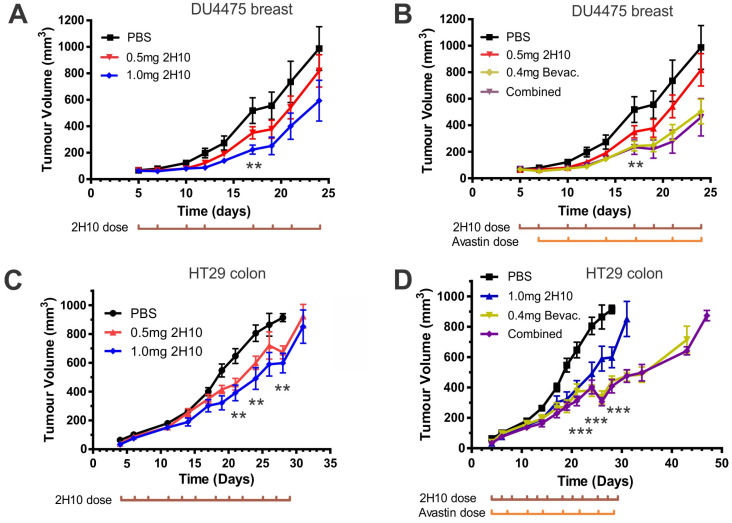
Tumor growth curves of xenografts from mice treated with anti-VEGF-B 2H10 or anti-VEGF-A bevacizumab, alone and in combination. (**A**,**B**) 2H10 treatment of mice with DU4474 breast carcinoma xenografts (*n* = 10), at the indicated doses: (**A**) alone (** *p* < 0.01 1 mg 2H10 v PBS); (**B**) in combination with 0.4 mg bevacizumab (Bevac.) (** *p* < 0.01 combined v PBS, *p* < 0.05 bevacizumab v PBS). 2H10 treatment 3×/week, bevacizumab 2×/week. (**C**,**D**) 2H10 treatment of mice with HT29 colon carcinoma xenografts (*n* = 12) at the indicated doses: (**C**) alone (** *p* < 0.01 0.5 mg or 1 mg 2H10 v PBS); (**D**) in combination with 0.4 mg bevacizumab (*** *p* < 0.001 bevacizumab or combined v PBS). Data are expressed as mean tumor volume ± standard error of the mean (SEM) at each time point.

We then treated mice bearing DU4475 breast xenografts with 2H10, compared to anti-human VEGF-A (bevacizumab) alone, or in combination. 2H10 treatment caused a significant, dose-dependent growth inhibition compared to vehicle control (PBS, Figure 3A,C), with a corresponding increase in survival to ethical endpoint (Supplementary Materials Figure S2A). Treatment with bevacizumab also inhibited tumor growth (Figure 3B), and extended survival (Figure S2A). Combination with 2H10 did not improve bevacizumab response, inferring overlapping mechanisms of inhibition. Similar results were obtained from treating mice with HT29 colon tumor xenografts (Figure 3C,D), or with MDA-MB-231 breast tumor xenografts (Supplementary Materials Figure S3).

**Figure 4:**
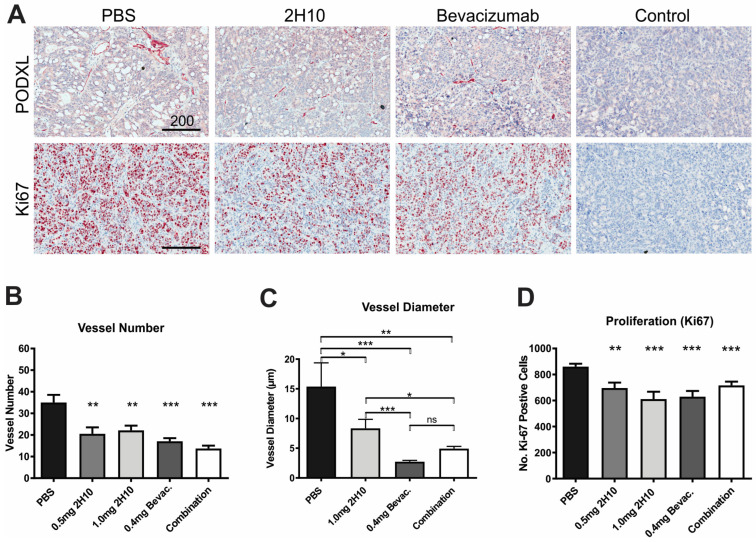
Immunohistochemical analysis of HT29 xenografts from anti-VEGF-treated mice. (**A**) Representative IHC images of matched HT29 tumor sections from PBS, 2H10- and bevacizumab-treated mice (from Figure 3), stained with anti-Podocalyxin for blood vessels, or anti-Ki-67 for proliferation, versus IgG controls. (**B**,**C**) Graphs show the average number of Podocalyxin-positive vessels (**B**) and vessel diameter (**C**) determined in ten regions/section. (**D**) Average number of Ki-67-positive nuclei. Graphs show means +/− SEM (ns = not significant, * *p* < 0.05, ** *p* < 0.01, *** *p* < 0.001).

To investigate the possible effects of treatment on tumor blood vessels, we analyzed HT29 xenograft samples by immunohistochemical (IHC) staining for the endothelial marker podocalyxin (Figure 4A). Both 2H10- and bevacizumab-treated samples exhibited a decrease in the number of vessels compared to control tumors (Figure 4B), as well as a decrease in the diameter of vessels (Figure 4C). This indicates the inhibition of neo-vascularization, as well as changes suggestive of structural normalization, which is characterized by morphological changes wherein tumor vessel diameters, density, and tortuosity are reduced [23]. Changes in vasculature were also accompanied by decreases in cell proliferation, assessed by Ki67 staining (Figure 4D). We also assessed tumors from control and 2H10-treated mice for changes in gene expression by Illumina Whole-Genome Gene Expression Array, both for human genes (tumor) and mouse genes (stroma). KEGG pathway analysis indicated that metabolic pathways were most significantly affected in tumor cells, while mouse gene changes included pathways associated with actin cytoskeleton, focal adhesion and hypoxic signaling, consistent with effects on the stroma and vasculature (Supplementary Materials Figure S4A,B).

### 2.4. VEGF-B Neutralisation Alters Intra-Tumoral Diffusion

**Figure 5:**
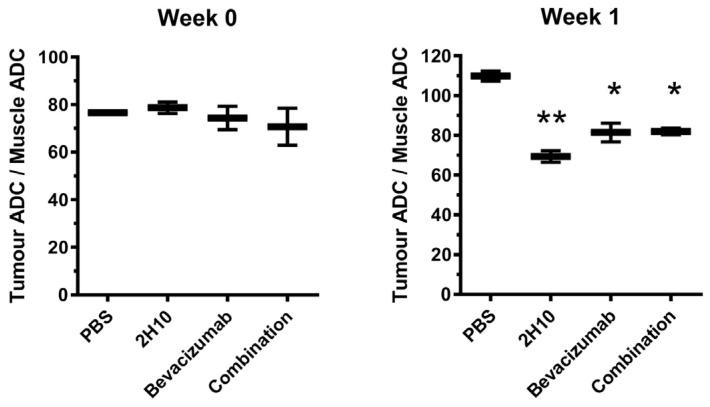
Comparison of apparent diffusion coefficients (ADC) in 2H10- and bevacizumab-treated or control tumors. The average ADC ratios in tumors relative to muscle were determined in mice with DU4475 xenografts before and after 1 week of treatment with 2H10 (1 mg), bevacizumab (0.4 mg), or both in combination (*n* = 2, mean with standard deviation). No significant difference was found between the four mice groups at Week 0. All treatment groups showed significantly reduced ADC ratio after one week of treatment relative to the control group (one-way ANOVA, * *p* < 0.05, ** *p* < 0.01).

Since anti-VEGF treatment resulted in alterations to the tumor vessel number and size, we next examined tumor perfusion. The apparent diffusion coefficient (ADC) is a measure of cellular integrity, where a high ADC reflects larger extracellular volume and fluid accumulation [24]. Hence, a smaller ADC can reflect vessel normalization and reduced leakiness, as well as increased cellularity. We determined ADC values from diffusion-weighted MRI imaging (DWI), to provide a physiological parameter of the diffusion of water within tumors, or skeletal muscle tissues as a matched normal tissue reference. Mice with DU4475 tumors were treated with 2H10 (1 mg), bevacizumab (0.4 mg), or both in combination (as above) and imaged at the start (week 0) and after one and two weeks. There was a significant decrease in tumor–muscle ADC ratios after one or two weeks of treatment with either 2H10 or bevacizumab, or both, compared to controls (Figure 5). This is consistent with known effects of anti-vascular therapies on intra-tumoral diffusion associated with vessel normalization [25].

We also used PET-based imaging approaches in mice with HT29 tumors to explore if 2H10 treatment resulted in detectable changes in tumor metabolism or hypoxia, as suggested above. Mice treated with 2H10 or bevacizumab, alone or in combination, were imaged for intratumoral glucose metabolism (^18^F-FDG) or hypoxia (^18^F-FMISO). No significant differences in the standardized uptake value (SUV) in treated tumors were observed, although this technique may not detect microenvironment changes in SUV. FDG PET indicated lower Metabolic Tumor Volume (MTV) and Total Glycolytic Activity (TGA) in all treated tumors, reflecting their much smaller size.

### 2.5. Anti-VEGF-B 2H10 Enhances Anti-Tumor Effects of Chemotherapy

**Figure 6:**
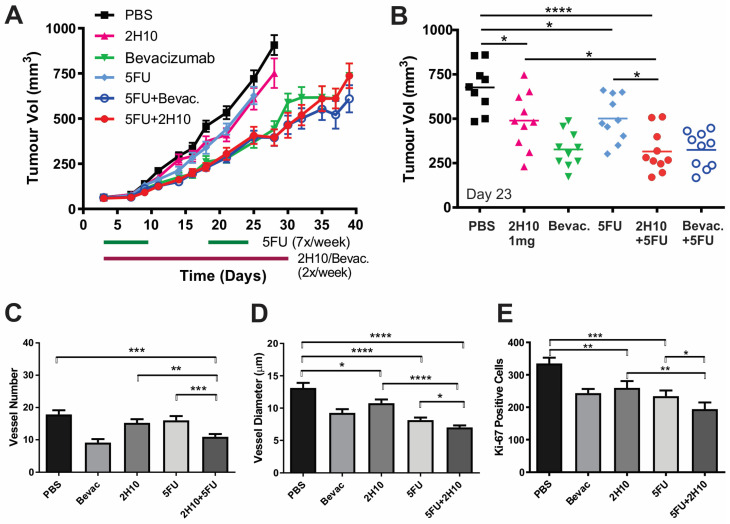
Combination of 2H10 with chemotherapy 5-FU. (**A**,**B**) Mice with HT29 tumors were treated with 2H10 (1 mg), bevacizumab (0.4 mg) or 5FU (20 mg/kg), alone and in combination as indicated. Tumor growth was monitored and growth curves plotted (**A**). (**B**) All data point at day 23. (**C**–**E**) Quantification of tumor IHC staining of markers for blood vessels (Podocalyxin) and proliferation (Ki-67). Graphs show vessel numbers (**C**) and diameter (**D**), and numbers of Ki-67-positive cells (**E**), in matched control and treated tumors (* *p* < 0.05, ** *p* < 0.01, *** *p* < 0.001, **** *p* < 0.0001).

Lastly, since vascular normalization can increase access and thus the therapeutic benefit of chemotherapy, we tested 2H10 and bevacizumab treatment in combination with Fluorouracil (5FU) (20 mg/kg) compared to each agent alone (Figure 6). Combined treatment of HT29 xenografts with 2H10 and 5FU resulted in significantly increased tumor inhibition compared to either treatment alone (Figure 6A,B), with a corresponding increase in survival analysis (Figure S2B). Bevacizumab treatment resulted in a similar degree of inhibition but was not further improved in combination with 5FU. Immunohistological staining of tumors indicated that 5FU and 2H10 combination therapy significantly decreased blood vessel number and diameter, and tumor cell proliferation, compared to either treatment alone, consistent with the observed inhibition of tumor growth (Figure 6C–E).

## 3. Discussion

Currently approved anti-angiogenic treatments focus on blocking VEGF-A-activated VEGFR activity, including via its best characterized receptor VEGFR2. However, VEGFR1 and its specific ligands VEGF-B and PlGF are also potential therapeutic targets. Indeed, significant anti-tumor efficacy has previously been reported in breast cancer xenograft models using VEGFR-1-neutralizing antibodies, targeting both tumor and stromal cells, and improving the response to chemotherapy [26,27]. Although initial phase 1 trials with anti-VEGFR-1 IMC-18F1 (Icrucumab) showed some potential anti-tumor activity [28], combination with the chemotherapy Capecitabine in patients with advanced or metastatic breast cancer did not significantly improve the response compared to chemotherapy alone [29]. While anti-VEGFR-1 antibodies were found to block VEGF-A and PlGF, the role of VEGF-B was not investigated.

This present study examined the suitability of targeting anti-VEGF-B through the novel, mouse/human cross-reactive antibody 2H10. We found that VEGF-B is expressed in tumor cells, stromal cells and blood vessels in a range of human tumor types, and biodistribution analysis in DU4475 breast cancer xenografts showed 2H10 selectively accumulated in tumors compared to normal tissues, from which the antibody more rapidly cleared. Furthermore, 2H10 was observed to have anti-tumor effects when given as monotherapy in both breast and colorectal cancer models. Consistent with previous findings targeting VEGF-A [30], we observed a decrease in vessel number and diameter using either bevacizumab or 2H10 as a monotherapy, accompanied by reduced proliferation in tumors, consistent with effects on tumor cells expressing VEGFR1. Combined treatment did not significantly improve the response compared to bevacizumab alone, suggesting the overlapping function of VEGF-A and VEGF-B, perhaps via their shared receptor VEGFR1.

Anti-angiogenic agents have the potential to normalize the tumor vasculature, reducing not only vessel density, but also abnormally dilated and leaky vasculature characteristically found in tumors, which can result in increased interstitial fluid pressure and metastasis [30]. Anti-angiogenic treatment can thus increase perfusion and provide a tumor microenvironment more susceptible to combined therapy [23,31,32,33,34]. Indeed, like bevacizumab, 2H10 treatment caused changes in tumor vascularity and intra-tumoral diffusion consistent with vascular normalization and, accordingly, 2H10 treatment improved the response to chemotherapy 5FU. This is in line with previous work showing that knockdown of VEGF-B in a high-expressing melanoma model resulted in increased perivascular cell coverage and decreased metastasis, whereas increasing VEGF-B in low-expressing models resulted in decreased pericyte coverage, leaky vasculature, tumor hypoxia and metastasis [19]. Interestingly, these effects appeared independent of VEGF-A and also of VEGFR1, suggesting the possibility of alternate mechanisms of VEGF-B in this context [19]. VEGF-B has been shown to have profound metabolic effects in several murine models of diabetes and diabetic complications [12,13,14,35]. This may suggest that at least some of the observed effects on tumor growth, structure of the tumor microenvironment, and the enhanced response to chemotherapy may be due to metabolic effects of the antibody treatment. This highlights the need for further investigation of VEGF-B in tumor biology, angiogenesis, and treatment, for which conformational specific antibodies such as 2H10 could play an important role.

## 4. Conclusions

In conclusion, we found that treatment of mouse breast and colon xenograft models with anti-VEGF-B antibody 2H10 resulted in reduced tumor growth, associated with decreased density and dilation of the tumor vasculature, and reduced fluid accumulation. Accordingly, 2H10 treatment improved the response to chemotherapy 5FU. This suggests that agents targeting VEGF-B may have therapeutic benefit, in addition to established treatments targeting VEGF-A, particularly in combination with chemotherapy.

## 5. Materials and Methods

### 5.1. Cell Lines and Antibodies

The DU4475 and MDA-MB-231 human breast adenocarcinoma cell lines, and the HT29 and COLO205 colorectal adenocarcinoma cell lines were obtained from ATCC (Manassas, MD, USA). All cell lines were cultured in RPMI/10% FCS and maintained at 37 °C in 10% CO_2_.

The murine parental IgG2a 2H10 antibody and the IgG1 reformatted 2H10 antibody, recognizing human and murine VEGF-B isoforms, and the IgG2a isotype control, were provided by CSL Ltd. (Parkville, VIC, Australia). Commercially available antibodies were obtained for human VEGFR-1 (rabbit polyclonal sc-9029 from Santa Cruz, Dallas, TX, USA, and 1527-PABX from Thermo Scientific, Scoresby, VIC, Australia) and VEGFR-2 (rabbit polyclonal sc-504, Santa Cruz, Dallas, TX, USA,; rabbit monoclonal antibody 55B11, Cell Signaling, Danvers, MA, USA). Secondary antibodies were FITC-labeled goat anti-rabbit IgG polyclonal antibody (ABR Affinity Bioreagents, Golden, CO, USA), and FITC-labeled goat anti-mouse IgG polyclonal (Calbiochem/Merck, Bayswater, VIC, Australia). Avastin (bevacizumab), a humanized anti-VEGF-A antibody (Roche/Genentech, San Francisco, CA, USA), specific for all human but not murine or rat VEGF-A isoforms [36], was provided by CSL Ltd.

### 5.2. Immunohistochemistry

Paraffin sections were deparaffinized and rehydrated and treated with 3% H_2_O_2_ for 10 min to quench endogenous peroxidase activity followed by a TBST (Tris-buffered saline Tween 20; 0.05 M Tris-HCl, 0.6% NaCl, 0.05% Tween 20, pH 7.6) wash. Primary antibodies or isotype controls were diluted in TBST at 10 µg/mL and applied to tissue sections for 120 min at 37 °C. Tissue sections were washed in TBST prior to incubation with secondary antibody (Dako Envision anti-mouse-HRP; 30 min 37 °C). Sections were stained with the chromogen AEC (3-amino-9-ethyl-carbazole; Sigma-Aldrich, St. Louis, MO, USA) for 20 min followed by counterstaining with Mayer’s haematoxylin. Tissue sections were permanently mounted with DePeX and a glass coverslip.

Xenograft tumors were removed surgically from mice, cut in half and either snap-frozen or fixed in 10% (*v*/*v*) formalin overnight. After paraffin embedding, 4 µm sections were cut and mounted. Sections were deparaffinized in a 60 °C oven followed by washes in xylene and ethanol. Tissue peroxidize activity was quenched by incubation in 3% (*v*/*v*) H_2_O_2_ in dH_2_O for 20 min at room temperature. Sections were treated using 10% citrate buffer, pH 6.0 (Labvision) using a 100 °C boiling water bath for 30 min to ‘retrieve’ antigens. After cooling, non-specific binding was blocked using Maxitage protein blocking agent (Thermo Fisher) at room temperature for 30 min. Slides were subsequently transferred into slide racks (Thermo Shandon) and developed using the appropriate primary antibodies as follows: Ki-67: sections were incubated with 1/100 rabbit anti-human Ki-67 (Thermo Scientific, RM-9106) for 2 h at room temperature, washed with PBS and incubated with anti-rabbit IgG-HRP (Dako, K4003) for 30 min. Staining was visualized following development with AEC for 20 min and Mayer’s hematoxylin staining. Podocalyxin: sections were incubated with 1ug goat anti-human podocalyxin (R&D, AF1556) antibody for 2 h at room temperature. Following PBS washing, sections were incubated with biotinylated anti-goat secondary and visualized using the Vectastain ABC kit (Vector Laboratories, Newark, CA, USA) according to manufacturer’s instructions. Analysis of treatment time-matched tumor sections was carried out using Aperio ScanScope XT in ten 500 µm × 500 µm regions/sections: anti-Podocalyxin-stained vessels were manually counted and vessel diameter was measured using the micrometre tool in the Aperio software, while Ki-67-positive cells were counted with the nuclei-counting tool.

### 5.3. Flow Cytometry Analysis

For intracellular staining cells were fixed with 70% ice-cold ethanol overnight then washed in PBS. Cells were permeabilized by incubation with FACS Lysing Solution (Becton Dickinson, Macquarie Park, NSW, Australia; 4 °C, 5 min). Permeabilized cells were stained with anti-VEGF-B 2H10 or MAB751 at 10 µg/mL and compared to staining with murine IgG_2a_ isotype control (10 µg/mL). Staining with anti-VEGFR-1 and anti-VEGFR-2 rabbit polyclonal antibodies at 10 µg/mL was compared to cells stained with secondary antibody alone. FITC-labeled secondary antibodies were diluted 1 in 100. Staining was assessed using a FACS Calibur flow cytometer and the FlowJo v7.5.5 software package.

### 5.4. Radiolabeling

Radiolabeling was performed on the day of injection into mice. Prior to injection, the percentage of unbound radionuclide content was determined by instant thin-layer chromatography, and the binding ability (immunoreactivity) of the final radiolabeled product was tested by binding to VEGF-B or control glycine-conjugated Sepharose beads in a modified Lindmo assay as detailed below.

The radiolabeling of murine 2H10 with Iodine-125 (^125^I; Perkin Elmer, Waltham, MA, USA) or Indium-111 (^111^In; Nordion, Ottawa, ON, Canada) was undertaken as previously described [37]. Briefly, the 2H10 antibody was trace labeled with ^125^I using the standard iodogen method and purified on a centrifugal desalting column. The 2H10 antibody was also labeled with ^111^In via the bifunctional metal ion chelate CHX-A″-DTPA (C-functionalized *trans*-cyclohexyl-diethylenetriaminepentaacetic acid) according to methods previously described [37]. The products were purified on a Sephadex G50 column (Pharmacia, Uppsala, Sweden) equilibrated with sodium chloride BP. For all radioconjugates, fractions with peak radioactivity were pooled and sterilized by passing through a 0.22 μm filter.

### 5.5. Radioactivity and Radiochemical Purity

Radioactivity was measured with an Atomlab-100 dose calibrator (Biodex, Brookhaven, NY, USA) or with a Cobra II automated gamma counter (Canberra-Packard, Melbourne, VIC, Australia). Radiochemical purity of labeled constructs was analyzed using an Instant Thin Layer Chromatography Silica Gel (ITLC-GC, Gelman Sciences, Inc., Ann Arbor, MI, USA) and developed using 10% *w*/*v* trichloroacetic acid as solvent.

### 5.6. Immunoreactivity

Radiolabeled 2H10 immunoreactivity was determined using a modification of the Lindmo assay [38]. Radiolabeled 2H10 was added to VEGF-B-conjugated or control glycine-conjugated Sepharose beads in 1.0 mL of medium or PBS. The mixture was incubated for 45 min at RT with continuous mixing to keep the beads in suspension. Beads were harvested by centrifugation and washed three times to remove unbound antibody. Radioactivity associated with beads or supernatant was measured in a gamma counter.

### 5.7. Serum Stability

Serum stability was assessed by incubating 5.0 µg of each radiolabeled protein in 200 µL of healthy donor human serum at 37 °C for an 8-day period. Radiochemical purity and single-point immunoreactivity assays at 0 (no incubation), 2 and 8 days of incubation were determined.

### 5.8. Animal Models

All animal experiments described in this study were approved by the animal ethics committee of the Austin Hospital (approval 2009/03381) and were conducted in compliance with the NHMRC guidelines “Australian code of practice for the care and use of animals for scientific purposes”. In vivo investigations were performed in 5–7 week old female athymic BALB/c nude mice, homozygous for the nu/nu allele bred by the Animal Research Centre, Perth, WA, Australia. Mice were maintained in autoclaved microisolator cages and housed in a positive pressure containment rack using an individually vented caging system (Techniplast IVC System, Milan, Italy). Tumor volume in mm^3^ was determined using the formula (length × width^2^) × 0.5, where length was the longest axis and width was the measurement at right angles to the length. Tumor growth curve data were expressed as mean tumor volume ± SEM (standard error of the mean) for each treatment group.

### 5.9. Biodistribution Study

Thirty BALB/c nude mice were used for the initial study examining the biodistribution of ^111^In- and ^125^I-radiolabeled murine 2H10 antibody in DU4475 tumor-bearing mice (15 days after subcutaneous left flank injection of 2 × 10^6^ DU4475 cells with matrigel in BALB/c nude mice). Groups of five mice were euthanized at 4, 24, 48, 72, 120 and 168 h (viz. 4 h, day 1, 2, 3, 5 and 7) following intravenous injection of a mixture of ^111^In-labeled murine 2H10 antibody (4.5 µg, 17.8 µCi) and ^125^I-labeled murine 2H10 antibody (4.8 µg, 7.3 µCi) via the tail vein (total volume 0.1 mL). At the commencement of the biodistribution/pharmacokinetic study, the mean tumor volume for DU4475 tumor xenografts was 70.39 ± 23.40 mm^3^ following 15 days of growth in mice, and mice were 7–8 weeks of age. For comparison of 2H10 isotypes, non-tumor-bearing mice were injected with a mixture of ^131^I-labeled murine 2H10 IgG1 and ^125^I-labeled murine 2H10 IgG2a antibodies with or without 1.1 mg unlabeled 2H10 (murine IgG2a) as a cold competitor. At the designated time points after injection of the radioconjugates, groups of mice (*n* = 5 for DU4475 tumor study; *n* = 4 for non-tumor study) were euthanized and exsanguinated by cardiac puncture and tumors and organs [liver, spleen, kidney, muscle, skin, bone (femur), lungs, heart, stomach, brain, small bowel, tail and colon] were resected immediately. All samples were counted in a dual gamma scintillation counter (Packard Instruments). Triplicate standards prepared from the original injected material were counted at each time point with tissue and tumor samples, enabling calculations to be corrected for the decay of each radioisotope. The tissue distribution data were calculated as the mean ± SD percent of injected dose per gram of tissue (%ID/g) at each time point.

### 5.10. Animal Therapy Studies

Xenografts were established by subcutaneous left flank injection on day 0 (2 × 10^6^ DU4475 or 5 × 10^6^ MDA-MB-231 cells in 150 µL PBS with 50% Matrigel (Becton Dickinson, Macquarie Park, NSW, Australia), or 2 × 10^6^ HT29 cells in PBS without Matrigel, as indicated). Therapy commenced once tumors had reached a mean volume of 50–100 mm^3^. Groups of 10 to 12 mice received intra-peritoneal (i.p.) injections of PBS vehicle control, or anti-VEGF-B 2H10 (IgG2a) (up to 1 mg as indicated) three times weekly, or 0.4 mg bevacizumab twice weekly, or a combination of both 2H10 and bevacizumab. Animals (*n* = 1 or 2) were sacrificed from each group after 1, 2 and 3 weeks of therapy. For chemotherapy treatments, 20 mg/Kg 5FU was administered every day on alternate weeks, alone or in combination with 2H10 or bevacizumab as indicated.

### 5.11. PET/MRI Imaging

All PET/MRI scans of the tumor-bearing mice were conducted using a nanoPET/MRI hybrid preclinical imaging system (Mediso, Budapest, Hungary) in tail-first supine position. At 67.27 ± 6.01 min before PET scan, mice were intravenously injected with 100 µL of radiotracer comprising 7.65 ± 0.64 MBq of ^18^F-FDG via tail vein. Each mouse was anesthetized using isoflurane via inhalation and maintained in a MINERVE anesthetic assembly. The mouse was then transferred to a single-mouse anesthesia/imaging closet chamber for MRI scans followed by a PET scan. The anesthesia/imaging chamber was integrated with heating and temperature control, as well as respiratory and ECG monitoring.

### 5.12. MRI

MRI scans were performed on the 1-Tesla static MRI sub-system which was equipped with a gradient of 450 mT/m strength and 35 mm diameter excitation and transceiver coils. The following parameters were used in the T1-weighted sequence: repetition time (TR) 25 ms, echo time (TE) 2.2 ms, transaxial FOV 48mm, and slice thickness 0.5 mm. The following parameters were used in the T2-weighted sequence: TR 4533 ms, TE 90 ms, transaxial FOV 50 mm, slice thickness 1.0 mm. The following parameters were used in the SE DWI sequence: TR 600 ms, TE 20.4 ms, FOV 50 mm, slice thickness 1.7 mm, and b values: 0 and 500 s/mm^2^.

### 5.13. PET

Following the MRI sequences, a 10 min static PET scan was acquired in list mode using the LYSO-based full ring PET sub-system with a maximum axial FOV of 120 mm. All PET raw data were decay, dead-time, random, and attenuation corrected and were reconstructed into volumetric images with a trans-axial matrix size of 255 × 255 using the built-in quasi Monte Carlo simulation algorithm combined with stochastic iteration and filtered sampling [39]. The size of the voxels in the reconstructed images was 0.4 mm × 0.4 mm × 0.4 mm.

### 5.14. Image Co-Registration and Tumor Delineation

The image co-registration and tumor delineation were performed in the Fusion Toolbox of PMOD 3.3 (PMOD technologies Ltd., Zurich, Switzerland). The generous contours of an entire tumor region were drawn free-hand on the consecutive trans-axial slices of the MRI T1W, T2W and ADC fused image and reviewed in the sagittal and coronal planes. The PET-based tumor contours were derived by refining the MRI-derived tumor contours on the co-registered PET image of the mouse. The potential impact of partial volume effects was also taken into account by excluding any anomalistic high uptake from the neighboring non-tumor regions.

### 5.15. Quantitative Image Analysis

The co-registered MRI/PET images and tumor contours were imported into MATLAB 7.4 (MathWorks Inc., Natick, MA, USA) for further image manipulation and analysis. ADC maps were calculated from the DWI images for all acquired slices using the standard equation:

(1) $$ADC({\mathrm{mm}}^{2}/s)=\frac{-In(S_{1}/S_{0})}{b_{1}-b_{0}}$$

where *S*_0_ and *S*_1_ are the signal intensities of a voxel in the diffusion weighted images after application of a diffusion gradient at *b*_0_ = 0 s/mm^2^ and *b*_1_ = 500 s/mm^2^, respectively. Diffusion sensitivity is determined by the difference between *b*_0_ and *b*_1_. In the original PET images, ^18^F-FDG uptakes in tissue were represented using radioactivity concentration (kBq/mL). These images were normalized for injected dose and mouse body weight and converted into standardized uptake values (*SUV*s), defined as:

(2) $$SUV\;(g/\mathrm{mL})=\frac{Tracer\;Concentration\;(\mathrm{kBq}/\mathrm{mL})}{\frac{InjctedDose\;(\mathrm{kBq})}{Body\;Weight\;(g)}}$$

In PET images, the MRI-derived tumor regions were firstly segmented from the background using the PMOD-derived tumor contours. The tumor regions were further segmented using the fixed-threshold method at 50% of the maximum *SUV* in the GTR. *SUVmin*, *SUVmean* and *SUVstd*, metabolic tumor volume (MTV) and total glycolytic activity (TGA) were then measured from the segmented tumor regions. MTV is defined as the volume of the segmented PET tumor regions using 50% of the maximum *SUV* in the GTR. TGA was determined by multiplying the MTV and its associated *SUVmean* [40].

### 5.16. Statistical Analysis

For multiple comparisons, one-way ANOVA was used unless otherwise stated. All analyses were carried out using Graphpad Prism version 6.03. Data are presented as the mean ± SEM, unless otherwise stated.

## Data Availability

Data is contained within the article or Supplementary Material.

## Supplementary Materials

The following supporting information can be downloaded at: https://www.mdpi.com/article/10.3390/cancers16101902/s1, Figure S1: Comparison of biodistribution of IgG1 and IgG2a versions of 2H10 in mice; Figure S2: Survival analysis of mice with Du4475 (A) or HT29 (B) xenografts treated with anti-VEGF antibodies; Figure S3: Treatment of mice with MDA-MB-231 breast carcinoma xenografts; Figure S4: Gene expression analysis of control and treated HT29 tumors; Table S1: Comparison of percentage of positive cells between tumor and normal tissues; Table S2: Summary of VEGF-B and VEGFR1 protein expression in human normal tissues; Table S3: Summary of VEGF-B and VEGFR1 protein expression in human tumor tissues.
