# Supplementary material for: An Anti-VEGF-B Antibody Reduces Abnormal Tumor Vasculature and Enhances the Effects of Chemotherapy
Source: Cancers (Basel). 2024 May 16;16(10):1902. doi: 10.3390/cancers16101902 (PMC11119922; doi:10.3390/cancers16101902)
Supplement: Supplementary file 1 [file cancers-16-01902-s001.zip › cancers-2971513-supplementary.pdf]

Supplementary Information

An Anti-VEGF-B antibody reduces abnormal tumor vasculature and enhances effects of chemotherapy

Peter W. Janes[1,2]*, Adam C. Parslow[1,2]*, Diana Cao[1], Angela Rigopoulos[1], Fook-Thean Lee[1], Sylvia J. Gong[3,4], Glenn A. Cartwright[1], Ingrid J. G. Burvenich[1,2], Ulf Eriksson[5], Terrance G. Johns[6,7], Fiona E. Scott[1], and Andrew M. Scott[1,2,4,8]

[1]Tumour Targeting Program, Ludwig Institute for Cancer Research and Olivia Newton-John Cancer Research Institute, Melbourne, Australia; [2]School of Cancer Medicine, La Trobe University, Melbourne, Australia; [3]School of Computing, Engineering and Mathematical Sciences, La Trobe University, Melbourne, Australia; [4]Department of Molecular Imaging and Therapy, Austin Health, Melbourne, Australia; [5]Division of Vascular Biology, Department of Medical Biochemistry and Biophysics, Karolinska Institutet, Stockholm, Sweden; [6]Oncogenic Signalling Laboratory, Telethon Kids Cancer Centre, Telethon Kids Institute, Nedlands, Australia; [7]University of Western Australia, Crawley, Australia; [8]Department of Medicine, University of Melbourne, Melbourne, Australia.

* Equal contribution

Table S1: Comparison of percentage of positive cells between tumor and normal tissues

	VEGF-B expression (2H10)	
	**Tumor**	**Normal**
Brain	0 – 50%<br>(+/++; 73%)	-ve (0%)
Breast	5 – >75%<br>(+/++; 64%)	25 – 50%<br>(+/++; 66%)
Bowel, large	0 – >75%<br>(+/++; 59%)	0 – 50%<br>(+/++; 83%)
Kidney	25 – >75%<br>(+/++; 21%)	25 – 50%<br>(+/++; 83%)
Liver	5 – 75%<br>(+/+++; 88%)	5 – 75%<br>(+/++; 50%)
Lung	5 – >75%<br>(+/++; 53%)	0 – 50%<br>(+; 86%)

Key: % of positive cells (intensity of staining; proportion of positive tissues expressed as percentage)

Table S2: Summary of VEGF-B and VEGFR1 protein expression in human normal tissues

Normal Tissue	Anti-VEGF-B 2H10	Anti-VEGF-B MAB751	Results	Anti-VEGFR1	Results
Adrenal Gland	6/11	8/11	+ to ++ cortex, medulla; +ve vessels	9/10	+ to +++ cortex, medulla; +ve vessels
Bladder, urinary	11/11	11/11	+ to ++ epith; +ve vessels; -ve stroma	11/11	+ to +++ epithelium; +ve vessels; occ stroma
Brain	0/12	4/13	-ve (or occ +ve) parenchyma; occ +ve vessels	12/12	++ parenchyma; occ. vessels
Bowel, Large	10/12	12/12	+ to ++ mucosal epith; weak vessels	12/12	+ to ++ mucosal epith and lamina propia; occ vessels
Breast	4/6	6/7	+ to ++ ducts; occ. stromal and vessels	9/9	+ to ++ duct epith; +ve vessels and occ stroma
Brown adipose	6/6	6/6	+ fat; +ve vessels (sm muscle and perivasc)	6/6	++ brown fat; +ve vessels
Heart	9/11	11/11	+ to ++ cardiac muscle; -ve vessels	11/11	+ to +++ cardiac muscle; +ve vessels
Kidney	10/12	10/10	+ to ++ tubules; -ve glomeruli and occ. vessels	10/10	+ to +++ tubules; occ vessels; -ve glomeruli
Liver	6/12	8/11	+ to ++ hepatocytes and ducts; -ve vessels & stroma	12/12	++ to +++ hepatocytes & ducts; occ vessels
Lung	12/14	12/14	+ Type I pneumocytes, occ. Type II; +ve vessels	14/14	+ Type I pneumocytes, occ. Type II; +ve vessels
Pancreas	11/11	11/11	+ to +++ ducts & islets; occ vess; - ve stroma	11/11	+ to +++ ducts, islets and acini; -ve vessels; occ stroma
Placenta	6/6	6/6	+ to ++ trophoblasts; +ve vess	6/6	+ to ++ trophoblasts; focal vessels
Prostate, BPH	9/12	11/12	+ to ++ glands; occ vess; -ve stroma	12/12	+ to +++ gland epith; +ve stroma and focal vessels
Skeletal Muscle	4/4	4/4	+ muscle; occ vessels	4/4	++ muscle and vessels
Spleen	11/11	11/11	+ve red and white pulp; +ve vess	11/11	+ve red and white pulp, more widespread in red pulp
Thyroid	9/11	10/11	+ to +++ foll epith; occ vess	8/10	+ to ++ foll epith
Tonsil	8/12	9/11	-ve lymphocytes +ve vessels	6/11	+ to ++ lymphocytes and vessels
Uterus	2/3	3/3	+ to ++ glands; -ve vess	2/2	+ to +++ gland epith; -ve stroma and vessels

Table S3: Summary of VEGF-B and VEGFR1 protein expression in human tumor tissues

Tumor Tissue	Anti-VEGF-B 2H10	Anti-VEGF-B MAB751	Results	Anti-VEGFR1	Results
Brain GBM	8/10	10/10	+ to ++ tum; occ vessels	10/10	+ to ++ tum; -ve vessels
Breast IDC	8/11	9/11	+ to ++ tum; -ve vessels and stroma	7/11	+ to ++ tum; occ vessels, +ve stroma
Breast ILC	9/14	14/14*	+ to +++ tum; occ vessels	14/14	+ to ++ tum; occ vessels and stroma
Colorectal Adenocarcinoma	15/15	15/15	+ to ++ tum; -ve vessels and stroma	15/15	+ to +++ tum; occ vessels and stroma
Kidney RCC	16/27	26/27*	+ to ++ tum; occ. vessels and stroma	27/28	+ to ++ tum; occ vessels
Liver HCC	5/24	13/24	+ to ++ tum; -ve vessels	24/24	++ to +++ tum; -ve vessels
Lung Adenocarcinoma	14/16	16/16	+ to ++ tum; -ve vessels and stroma	16/16	++ to +++ tum; occ vessels and stroma
Lung SqCC	8/13	11/13*	+ to ++ tum; occ. vessels and stroma	12/12	+ to +++ tum; occ vessels, +ve stroma
Mesothelioma	9/19	14/19	+ tum; -ve vessels and stroma	17/17	+ to +++ tum; -ve vessels and occ stroma
Metastatic Melanoma	4/13	13/13*	+ to ++ tum; -ve vessels and stroma	13/13	+ to +++ tum; occ vessels, focal stroma
Ovarian Adenocarcinoma	11/15	12/15	tumor; occ. vessels and stroma	15/15	+ to +++ tumor; occ. vessels and stroma
Pancreatic Adenocarcinoma	6/8	6/8	+ to ++ tum; occ. vessels and stroma	5/8	+ to +++ tum; focal vessels, occ stroma
Prostate Adenocarcinoma	9/9	9/9	+ to ++ tum; occ vessels	9/9	+ to +++ tum; focal vessels and stroma
Uterine Adenocarcinoma	9/14	12/14	+ to ++ tum; -ve vessels and stroma	14/14	+ to +++ tum; -ve vessels and stroma

Figure S1. Comparison of biodistribution of IgG1 and IgG2a versions of 2H10 in mice

A mixture of ^{131}I-labeled murine 2H10 IgG1 and ^{125}I-labeled murine 2H10 IgG2a antibodies was administered to non-tumor-bearing balb/c nude mice alone (Figure S1 A,B) or with 1.1 mg unlabeled 2H10 (murine IgG2a) as a cold competitor (Figure S1 C,D). Biodistribution was measured at 3 hours (Figure S1 A,C) and 24 hours (Figure S1 B,D).

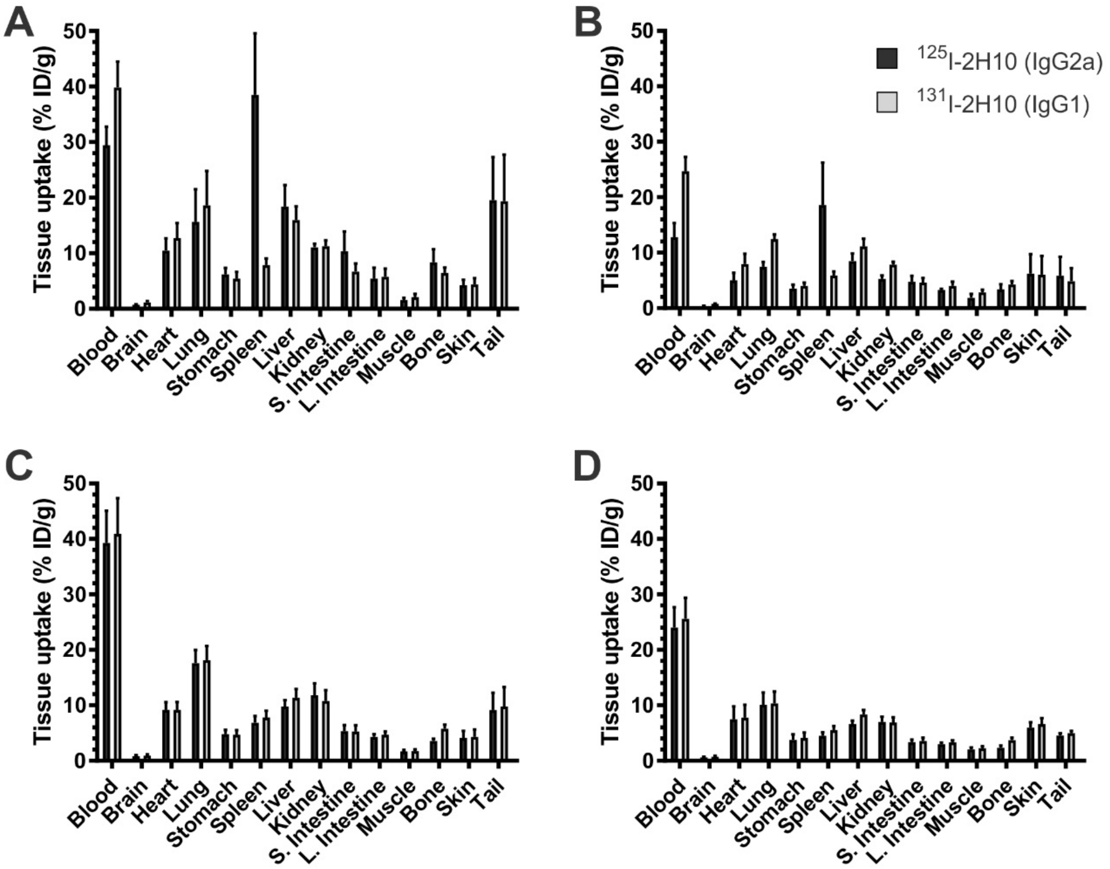

Figure S2. Survival analysis of mice with Du4475 (A) or HT29 (B) xenografts treated with anti-VEGF antibodies.

Graphs show survival of mice from experiments depicted in Figures 3 and 6, defined by reaching a set tumor volume threshold or ethical endpoint. Significant difference of curves was determined by Log-rank (Mantel-Cox test).

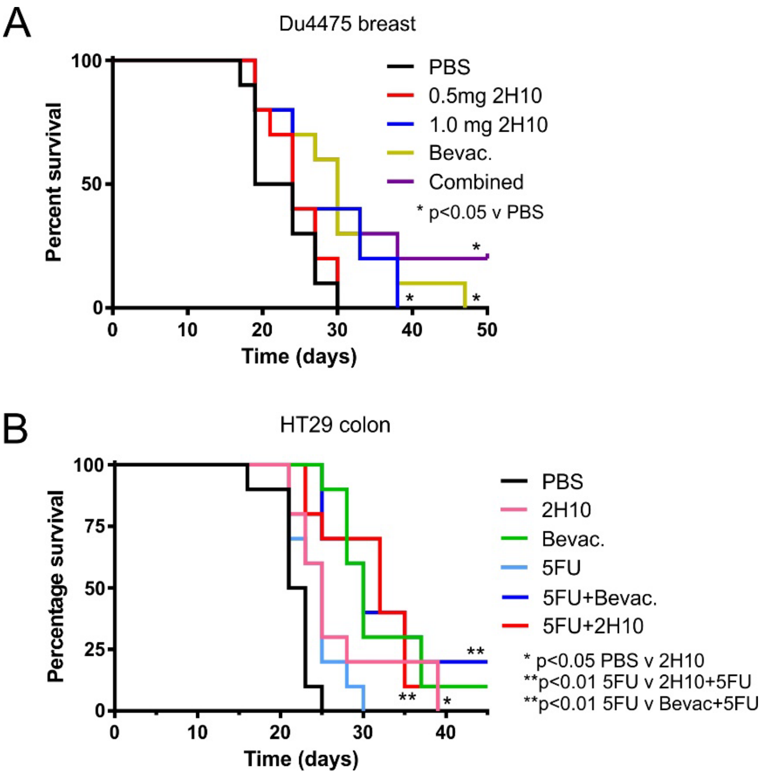

Figure S3. Treatment of mice with MDA-MB-231 breast carcinoma xenografts.

Mice bearing MDA-MB-231 tumors (n= 10) were treated at the indicated doses of 2H10, alone (A) or in combination with 0.4mg bevacizumab (B). 2H10 treatment 3x/week, bevacizumab 2x/week (*p<0.05 compared to PBS vehicle control).

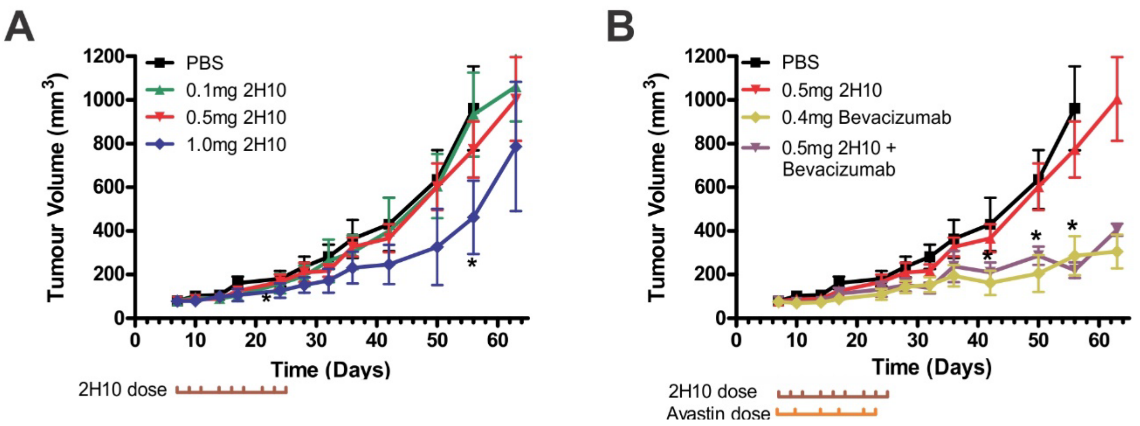

Figure S4. Gene expression analysis of control and treated HT29 tumors.

Mice with HT29 tumors were treated with 2H10 versus vehicle control (n=4) for 2 weeks. Tumors were recovered and cDNA samples were prepared and screened using the Illumina Human HT-12 v4 and Mouse WG6v2 Expression Bead chip arrays containing over 43,000 gene probes (Australian Genome Research Facility). Heat maps show hierarchical clustering of samples by gene expression changes, with up- and down-regulated genes shown in red and blue, respectively. Enrichment of key pathways are listed below.

A. Human (tumor) gene expression changes, 2H10 (II) v PBS.

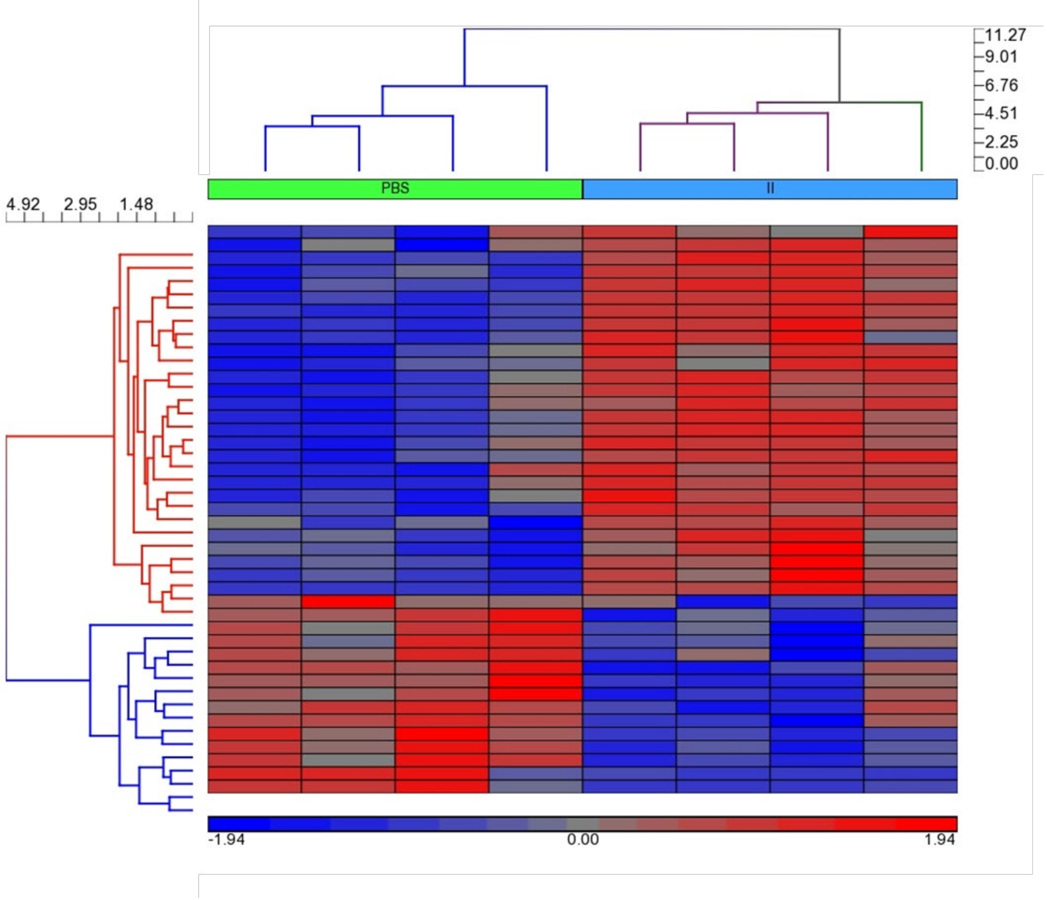

Pathway Name	Enrichment Score	Enrichment p-value
Citrate cycle (TCA cycle)	5.999	0.002
Lysine degradation	5.032	0.007
Arginine and proline metabolism	4.738	0.009
Sulfur metabolism	3.361	0.035
Lysosome	3.302	0.037
Metabolic pathways	2.887	0.056

B. Mouse (stroma) gene expression changes, 2H10 (II) v PBS.

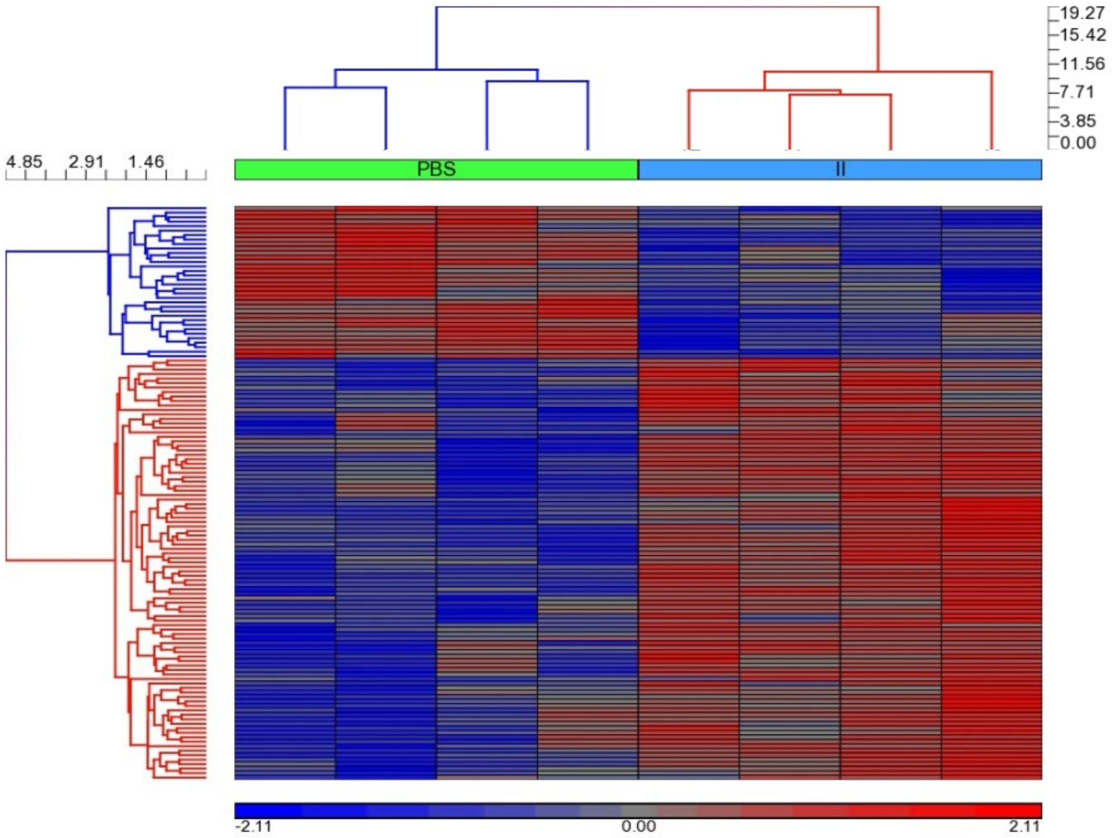

Pathway Name	Enrichment Score	Enrichment p-value
Regulation of actin cytoskeleton	5.896	0.003
Fc gamma R-mediated phagocytosis	5.849	0.003
HIF-1 signaling pathway	5.255	0.005
Pancreatic cancer	4.720	0.009
Glycosylphosphatidylinositol(GPI)-anchor biosynthesis	4.582	0.010
Bacterial invasion of epithelial cells	4.554	0.011
Focal adhesion	4.532	0.011
Arrhythmogenic right ventricular cardiomyopathy	4.437	0.012
Viral carcinogenesis	4.188	0.015
Base excision repair	3.832	0.022
Natural killer cell mediated cytotoxicity	3.031	0.048
